# Environmental and socio-demographic associates of children’s active transport to school: a cross-sectional investigation from the URBAN Study

**DOI:** 10.1186/1479-5868-11-70

**Published:** 2014-06-02

**Authors:** Melody Oliver, Hannah Badland, Suzanne Mavoa, Karen Witten, Robin Kearns, Anne Ellaway, Erica Hinckson, Lisa Mackay, Philip J Schluter

**Affiliations:** 1 Human Potential Centre, Auckland University of Technology, Private Bag 92006, Auckland 1142, New Zealand; 2 University of Melbourne, Melbourne, Australia; 3 Massey University, Auckland, New Zealand; 4 University of Auckland, Auckland, New Zealand; 5 Medical Research Council, Glasgow, Scotland; 6 University of Canterbury, Christchurch, New Zealand

**Keywords:** Built environment, Walkability, Walking, Cycling, Transport, Distance, New Zealand

## Abstract

**Background:**

Active transport (e.g., walking, cycling) to school (ATS) can contribute to children’s physical activity and health. The built environment is acknowledged as an important factor in understanding children’s ATS, alongside parental factors and seasonality. Inconsistencies in methodological approaches exist, and a clear understanding of factors related to ATS remains equivocal. The purpose of this study was to gain a better understanding of associates of children’s ATS, by considering the effects of daily weather patterns and neighbourhood walk ability and neighbourhood preferences (i.e., for living in a high or low walkable neighbourhood) on this behaviour.

**Methods:**

Data were drawn from the Understanding Relationships between Activity and Neighbourhoods study, a cross-sectional study of physical activity and the built environment in adults and children in four New Zealand cities. Parents of participating children completed an interview and daily trip diary that assessed their child’s mode of travel to school, household and individual demographic information, and parental neighbourhood preference. Daily weather data were downloaded from New Zealand’s national climate database. Geographic information systems-derived variables were calculated for distance to school and neighbourhood walkability. Bivariate analyses were conducted with ATS and potential associates; factors related to ATS at p < 0.20 were considered simultaneously in generalized estimation equation models, and backwards elimination of non-significant factors was conducted; city was treated as a fixed effect in all models.

**Results:**

A total of 217 children aged 6.5-15 years participated in this study. Female sex, age, city, household income, limited/no car access, residing in zone of school, shorter distance to school, neighbourhood self selection, rainfall, and sunlight hours were simultaneously considered in multivariate generalised estimation equation modelling (all p < 0.20 in bivariate analyses). After elimination of non-significant factors, age (p = 0.005), shorter distance to school (p < 0.001), city (p = 0.03), and neighbourhood self selection (p = 0.04) remained significantly associated with ATS in the multivariate analysis.

**Conclusion:**

Distance to school is the prevailing environmental influencing factor on children’s ATS. This study, in conjunction with previous research, suggests that school siting is likely an important associate of children’s ATS.

## Introduction

Active transport to school (ATS) is an important contributor to overall physical activity levels [1-3], maintenance of a healthy weight [4,5], and improved cardiovascular risk profiles [6] in children and young people. Shifting from motorised to active travel modes also has numerous social, economic, and ecological advantages [7-9]. Despite these benefits, declines in ATS have been observed in industrialised nations internationally [10-13]. In part, urban form changes which encourage motor vehicle use have been suggested as contributing to these declines in ATS [9].

The built environment is increasingly being acknowledged as having the potential to encourage sustained behaviour change for all members of society [9,14,15]. Inconsistencies in measurement approaches of both ATS and the built environment have hindered a clear understanding of the relationship between these factors [16]. Frank et al. [17] developed a neighbourhood walkability index (a combined measure of street connectivity, dwelling density, land use mix, and retail floor area ratio) to provide researchers with a systematic method for examining relationships between the built environment and physical activity. Subsequently a range of walkability indices and definitions (e.g., density and connectivity; density, connectivity, and land use mix; urban versus suburban environments) have been linked with active transport and physical activity in adults [18-20] and adolescents [21]. This relationship, however, is not well understood for children and young people. For example, differential relationships have been found by socio-economic status, with walkability associated with increased ATS in children residing in high income but not low income neighbourhoods [22]. Giles-Corti et al. [23] developed a more sophisticated ‘school walkability index’ by adding a measure of traffic exposure to the neighbourhood walkability index, and applied the measure at 2 km (1.2 mile) buffers around primary schools. Children attending schools located in highly walkable areas were 3.63 times more likely to walk to school than those attending schools sited in low walkability settings (95% CI 2.01-6.56). However, traffic volume mediated this relationship, whereby children living in areas with high street connectivity and high traffic volume were significantly less likely to walk to school (OR 0.32, 95% CI 0.22-0.47).

Other built environment factors have also been associated with ATS, including distance and connectivity [24] (although Trapp et al. [25] found this for boys but not girls), road density [26], and higher land use mix [27]. Distance to school is widely recognised as the prevailing urban form factor associated with reduced ATS [8,16,28-30]. Moreover, the magnitude of the effect of distance to school is substantial [31]; McDonald [32] showed that travel time had the strongest effect on ATS, whereby a 1 minute or 10% increase in walking time was associated with a 0.2% and 7.5% decline in likelihood of walking to school, respectively.

Notwithstanding the need for supportive urban form in the first instance, it is likely that parental directives are also associated with children’s ATS [22]. Parent-reported neighbourhood walkability, attitudes towards travel modes, traffic and ‘stranger’ safety concerns, and social support have all been linked with children’s ATS [33]. When questioned on specific barriers to ATS, the greatest factors cited by a sample of United States (US) parents were distance to school (61.5%), traffic danger (30.4%), and weather (18.6%) [34]. Similarly, bad weather was cited by nearly a third of US parents as a key reason for driving their child to school, after trip-chaining and backpack weight [24]. Interestingly, differential relationships were found by distance to school, whereby those who lived 1.5 miles or more from school were less likely to cite weather as influencing transport mode choice. A Canadian study reported that the greatest reason that parents continued to drive their child to school after a travel plan intervention was weather (21%), followed by convenience, trip chaining, and distance to school [35]. Conversely, Mitra and Faulkner [36] found that ATS was not associated with season or objectively-assessed weather (weekly precipitation days, snow days, average temperature) in Canadian children aged 11–12 years. It is possible this was because of homogeneity in distance from school (79.8% lived within 1.6 miles of school) and ATS behaviours (62.7%), or because the use of weekly weather factors did not allow for variability in weather and associated behaviours across days. Daily weather patterns (rainfall, temperature, sun hours) have been linked with physical activity in children in New Zealand [37,38] and the UK [39], however it is not clear whether this effect persists for ATS. The effect of weather on transport mode choice is not well understood, largely because proxy measures are usually employed, such as season, or aggregate measures of weather over the measurement period. Although non-modifiable, understanding the potential relationship between weather patterns and ATS is important; significant infrastructural and financial investment is made to encourage active travel modes to school internationally (e.g., school travel plans, safe routes to school) [40-43]. Therefore, it is essential to ensure such interventions account for weather conditions (e,g., providing umbrellas for walking school buses, implementing cycle skills and safety training specifically for inclement weather conditions) where associations exist between weather and school travel mode.

Not only are parents the gatekeepers to the ATS behaviours of their child [44,45], they also appear to determine the neighbourhood environment in which the child lives. Neighbourhood residential choice, also known as neighbourhood self-selection (NHSS) may be influenced by factors associated with ‘place’ such as residing in school zoning/catchment areas, distance to work, access to public transport, and housing affordability. These are intrinsically linked with ‘people’ factors such as employment and socioeconomic status, family structure, and mobility needs [46-48]. Those preferring to live in urban (more walkable), rather than suburban (less walkable) neighbourhoods are more likely to engage in work-related active transport modes, regardless if they actually live in high or low walkable environments [49]. NHSS is an emerging focus area in health and place-based research, and as such it remains unknown whether parental NHSS status (e.g., preference for, and living in, a high or low walkable neighbourhood) extends to influencing children’s ATS behaviours. Accordingly, the purpose of this study was *to build on existing research on associates of children’s ATS, by considering the associations between daily weather patterns and neighbourhood walkability and preferences (NHSS status) with ATS.*

## Methods

### Protocol

Data were drawn from the Understanding Relationships between Activity and Neighbourhoods (URBAN) study; complete methodology of all aspects of this larger study has been provided elsewhere [50]. Briefly, this was a multi-city, stratified, cross-sectional study of associations between physical activity, health, and the built environment in adults and children residing in New Zealand. Participants were recruited randomly from 48 neighbourhoods (stratified by high/low walkability, high/low Māori (New Zealand indigenous population)) across four New Zealand cities. Neighbourhoods were defined as being five contiguous meshblocks or more of similar walkability and Māori population density. A meshblock is a geographic census unit of approximately 100 households constructed for enumeration and analysis purposes by Statistics New Zealand [51]. Neighbourhood walkability was calculated using Geographic Information Systems (GIS)-derived street connectivity, dwelling density, land use mix, and retail floor area ratio at the mesh-block level. Summary scores (average of the mesh-block level walkability values) were calculated for each neighbourhood and neighbourhoods were partitioned into walkability tertiles (low/medium/high). In the interests of attaining maximal variability, only meshblocks with low (deciles 1–3) and high (deciles 7–10) walkability and Māori residential density were considered.

A door-to-door recruitment strategy was utilised, where every n^th^ household within a neighbourhood was sampled. The sampling rate was determined by density of dwellings within the neighbourhood, assuming a 60% response rate. One usually resident adult (aged 20 – 65 years) and child (aged 3–18 years) in each household were invited to participate. Eligibility criteria were: within the age range, English speaking, able to walk without aids (for physical activity measurement), and having resided in the household at least three months prior to, and for the week during, the measurement period. Children were only eligible to participate if there was a participating adult in the household. Where there was more than one eligible adult or child, the individual(s) with the next birthday were recruited. Children and youth aged 6–15 years were included in analyses for the current study.

Adults completed a 40-minute computer-assisted personal interview with a trained interviewer. The interview assessed individual and household demographics, neighbourhood perceptions and preferences, physical activities, and sedentary behaviours. Participants also completed a trip diary for the previous seven days including primary travel mode to and from school or work for each day. Adults completed the interview and trip diary on behalf of their child. The latter included data on the child’s primary travel mode to and from school each day. GIS measures of the built environment were determined using ArcView v 9.2 software (ESRI, Redlands, CA). Data were collected between April 2008 and August 2010, with some crossover between the four cities as follows: North Shore City, April 2008-April 2009; Waitakere City, November 2008-October 2009; Wellington City, May 2009-March 2010; Christchurch City, November 2009-September 2010.

All participants provided informed written consent. Ethical approval to conduct the study was provided by the host institutions’ ethics committees (Auckland University of Technology Ethics Committee reference number 07/126, Massey University Human Ethics Committee reference number 07/045). Measures specific to the current study are detailed below.

### Measures

#### Child measures

##### Active transport to school

Trips were coded as walking, cycling, private motorised transport, or public transport for every day of school attendance over the seven day measurement period using trip diary information provided by parents for travel mode to school. A binary variable was generated for ATS from these data (walking or cycling versus motorised transport).

##### Child demographics

Parents reported their child’s sex, ethnicity, and date of birth. In cases where multiple ethnicities were recorded, the priority system of Statistics New Zealand was employed (in the following order: Māori, Pacific, Asian, other European, New Zealand European) [52]. Child age was calculated from the date of birth to the date of survey completion, and classified as 5–10 years of age, 11–12 years of age, or 13 years of age or older (approximating to primary school years 1–6, intermediate school years 7–8, and secondary school years 9–13 in the New Zealand school system, respectively).

#### Environmental measures

##### Distance to school

Participants’ home and school addresses were geocoded and the closest facility function used to model the shortest street network commute between participants’ home and school address. Distance to school was classified into 0-700 m (0–0.4 miles), 701-1000 m (0.4-0.6 miles), 1001-2000 m (0.6-1.2 miles), or greater than 2000 m from home, roughly representing quartiles of these data.

##### Residing outside school zone

School zone information was obtained from the Ministry of Education and schools were identified as zoned or un-zoned. Enrolment schemes for New Zealand schools include a clearly defined boundary (school zone) in which residing children have an absolute right to enrolment at that school. Children living outside a specified zone for their chosen school are not guaranteed a place at that school. Participants who attended zoned schools were assessed as residing either within zone boundaries (in-zone) or outside zone boundaries (out-of-zone). Residing in-zone or attending an un-zoned school were combined, resulting in a dichotomous variable of residing outside school zone versus residing within school zone.

##### Weather

**Figure 1:**
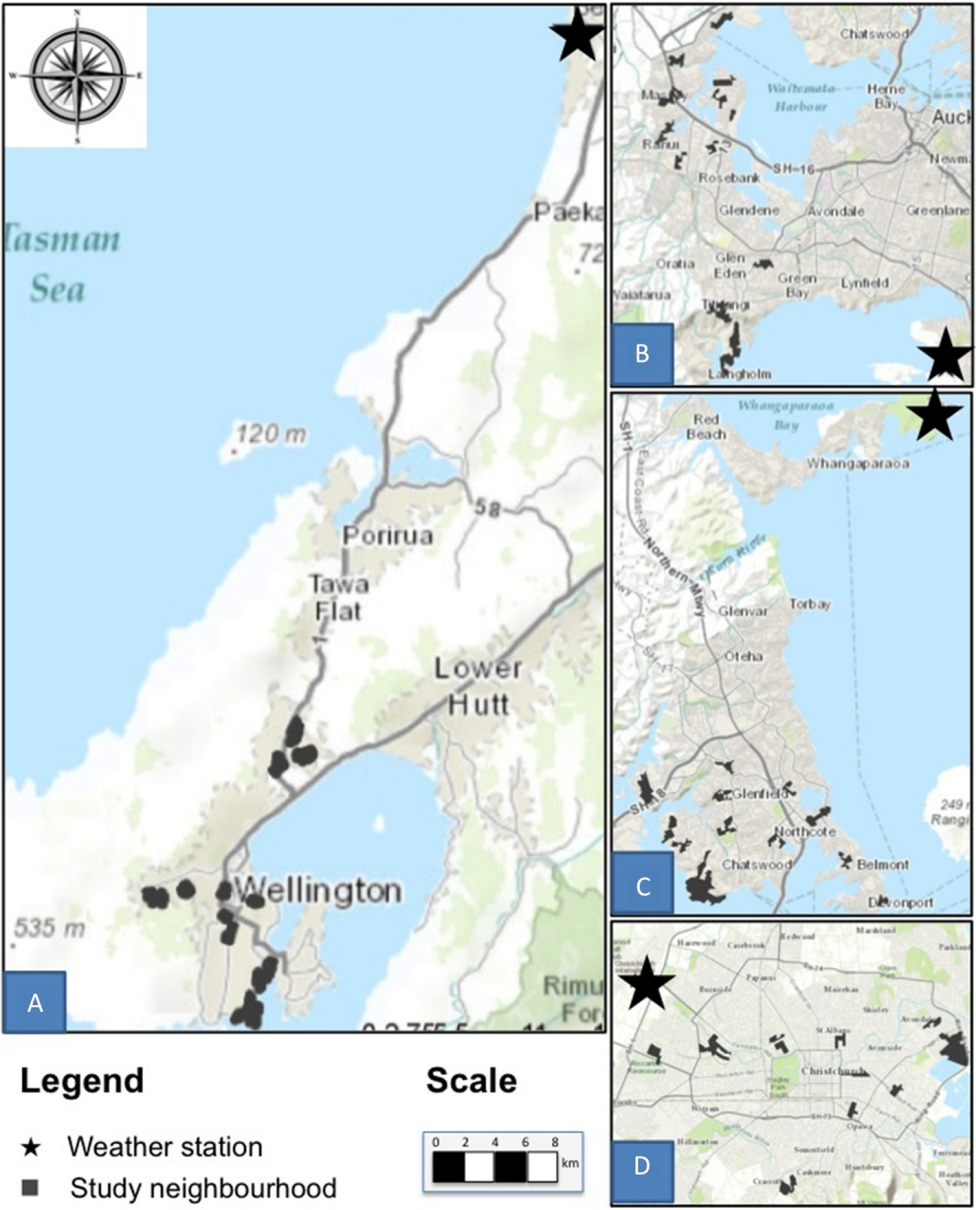
**Locations of study cities and weather stations within each city.** Note: **A** = Wellington City, **B** = Waitakere City, **C** = North Shore City, **D** = Christchurch City

Sunlight (hours), total rainfall (mm), and average temperature (degrees celsius) for each day were obtained from the national climate database for New Zealand (data are freely available from http://cliflo.niwa.co.nz/). Weather data for the climate database are sourced from Meteorological Service of New Zealand Limited (MetService) weather stations across the country. The database is maintained by the National Institute of Water and Atmospheric Research (NIWA). Daily summary data were extracted from the NIWA climate database for Whangaparaoa AWS (1400) for North Shore City, Mangere EWS (22719) for Waitakere City, Paraparaumu Aero AWS station (8567) for Wellington City, and Christchurch Aero (4843) for Christchurch City. Maps of the study cities and respective weather stations are provided in Figure 1.

#### Household and parent measures

##### Neighbourhood self-selection

Neighbourhood preference was assessed using items developed by Levine et al. [53] and as described in detail elsewhere [49]. Adult participants were asked whether they would prefer to live in a more suburban (less walkable) or urban (more walkable) environment, assuming housing cost, quality of schools, and mix of people were constant across neighbourhood type. Illustrations of neighbourhood types were shown to participants concurrent with detailed verbal descriptions of neighbourhood types. Neighbourhood walkability was defined as high or low, as described earlier. Preliminary analyses revealed a non-collinear interaction between neighbourhood preference and neighbourhood walkability in that the association between walkability and ATS only occurred when participants indicated a preference for a highly walkable neighbourhod (details available on request). NHSS status was classified using a combination of neighbourhood walkability and neighbourhood walkability preference [54] as follows: ‘prefer high walkable, live low walkable’, or ‘otherwise’ (i.e., prefer low walkable, live high or low walkable; or prefer high walkable, live high walkable), hereafter termed NHSS^PHLL^ and NHSS^OTH^, respectively.

##### Car access

Parents were asked to state whether they had ‘unrestricted access’, ‘frequent access’, ‘limited access’, or ‘no access’ to a personal motorised vehicle in the last week. Due to low numbers in the ‘frequent’ and ‘no access’ categories (Table 1), car access was dichotomised as unrestricted/frequent versus limited/none.

**Table 1:** Participant characteristics

	**Boys (n = 111)**		**Girls (n = 105)**		Total (n = 217)*	
	**n**	**(%)**	**n**	**(%)**	**n**	**(%)**
Age (years)						
5-10	44	(39.6)	40	(38.1)	85	(39.2)
11-12	27	(24.3)	37	(35.2)	64	(29.5)
13-14	40	(36.0)	28	(26.7)	68	(31.3)
Ethnicity						
Māori	25	(22.5)	27	(25.7)	53	(24.4)
Asian	20	(18.0)	13	(12.4)	33	(15.2)
New Zealand European/other	66	(59.5)	65	(61.9)	131	(60.4)
Average annual household income (NZD)						
<$20,000	8	(7.1)	5	(4.8)	13	(6.0)
$20,001-$40,000	19	(17.1)	14	(13.3)	33	(15.2)
$40,001-$60,000	21	(18.9)	18	(17.1)	39	(18.0)
$60,001-$80,000	14	(12.6)	13	(12.4)	27	(12.4)
$80,001-$100,000	12	(10.8)	15	(14.3)	27	(12.4)
>$100,000	21	(18.9)	32	(30.5)	54	(24.9)
Car access						
Unlimited	93	(83.8)	94	(89.5)	188	(86.6)
Frequent	5	(4.5)	3	(2.9)	8	(3.7)
Limited	9	(8.1)	4	(3.8)	13	(6.0)
None	4	(3.6)	4	(3.8)	8	(3.7)
City						
North Shore	23	(20.7)	21	(20.0)	45	(20.7)
Waitakere	36	(32.4)	32	(30.5)	68	(31.3)
Wellington	25	(22.5)	24	(22.9)	49	(22.6)
Christchurch	27	(24.3)	28	(26.7)	55	(25.4)
School zoning						
Residing in school zone	57	(62.0)	47	(50.5)	104	(55.9)
Residing outside school zone	15	(16.3)	16	(17.2)	32	(17.2)
No school zone specified	20	(21.7)	30	(32.3)	50	(26.9)
Distance to school						
0-700 m	26	(23.4)	27	(25.7)	53	(24.4)
701-1000 m	32	(28.8)	22	(21.0)	54	(24.9)
1001-2000 m	24	(21.6)	26	(24.8)	50	(23.0)
>2000 m	29	(26.1)	30	(28.6)	60	(27.7)
Neighbourhood self-selection						
Prefer high walkable, live low walkable	36	(32.4)	29	(27.6)	65	(30.5)
Prefer high walkable, live high walkable	21	(19.3)	22	(21.4)	43	(20.2)
Prefer low walkable, live low walkable	32	(29.4)	32	(31.1)	65	(30.5)
Prefer low walkable, live high walkable	20	(18.4)	20	(19.4)	40	(18.8)

*Sex data were missing for one participant; total number of participants was 217.

m, metres; n, number of participants; NZD, New Zealand Dollars; SD, standard deviation.

##### Socio-economic status

Respondents were asked to classify their combined annual household income as “none”, <$20,000, $20,001-$40,000, $40,001-$60,000, $60,001-$80,000, $80,001-$100,000, or > $100,001, in New Zealand dollars. Annual income was dichotomised as 0-$80,000 or greater than $80,000. The median annual household income for New Zealand in 2010 was $75,700 [55].

### Analyses

ATS was treated as a repeated measure for each school day. Preliminary crude (bivariate) analyses were first conducted for daily ATS and potential predictor factors. Factors were simultaneously considered in a binomial generalized estimation equation (GEE) model, clustered by child (assuming exchangeable correlation structures), and with the logit link function and Huber-White sandwich estimate of variance specified. Factors with Wald’s p-value < 0.20 in the bivariate analyses were entered into a multivariate GEE model and backward elimination of non-significant terms was conducted until the most parsimonious multivariate model was found [56]. City was specified as a fixed effect in the model and retained irrespective of statistical significance in the bivariate and multivariate analyses. Statistical significance was set at α = 0.05 and analyses were undertaken using Stata IC version 10.1 (StataCorp, TX, USA).

## Results

Across the four cities, a total of 217 children were recruited from 43 of the possible 48 neighbourhoods (12 per city); between 1 and 17 children were recruited within each of these neighbourhoods (Table 2). Because children were not recruited through schools, there was not a direct match between neighbourhood and school, and it is possible that children from multiple neighbourhoods attended the same school(s). There were a total of 101 different schools that children attended; between one and twelve children attended each school, with a median of one child per school found across all study cities (Table 2).

**Table 2:** Area-level characteristics

**City**	**Participants (n = 217)**		**Neighbourhoods (n = 43)**			**Schools (n = 101)**		
	**n**	**(%)**	**n**	**Median**	**(Range)**	**n**	**Median**	**(Range)**
North Shore	45	(20.7)	10	4	(1, 10)	25	1	(1, 7)
Waitakere	68	(31.3)	11	5	(1, 16)	28	1	(1, 12)
Wellington	49	(22.6)	10	5	(1, 17)	21	1	(1, 9)
Christchurch	55	(25.4)	12	4	(1, 10)	27	1	(1, 8)

n, number of participants, neighbourhoods, or schools.

Data on travel mode to school were available for 776 trips, as detailed in for each city in Table 3. A majority of trips were made by private motor vehicle (70% overall); cycle trips were the least prevalent mode of travel (1% of trips overall, 4% of ATS) and so these were combined with walking trips to generate an overall measure of ATS. Daily weather data are summarised for each city and overall in Table 4. Average daily temperatures ranged from 4.4-22.4 degrees celsius (mean = 13.2 degrees celcius), and sun hours ranged from 0–14.4 hours (mean 5.7 hours). Daily rainfall ranged from 0-47 mm; these data were highly skewed (Shapiro-Wilk p < 0.001), so were classified as some (n = 352, 45%) versus none (n = 424, 55%).

**Table 3:** Trip characteristics

**City**	**Total trips**	**Walk (n = 183; n = 42 children)**		**Cycle (n = 7, n = 4 children)**		**Private motorised transport (n = 539; n = 119 children)**		**Public transport (n = 47; n = 14 children)**	
	**n**	**n**	**(%)**	**n**	**(%)**	**n**	**(%)**	**n**	**(%)**
North Shore	198	67	(33.8)	3	(1.5)	110	(55.6)	18	(9.1)
Waitakere	170	46	(27.1)	0	(0)	123	(72.4)	1	(0.6)
Wellington	189	36	(19.1)	2	(1.1)	124	(65.6)	27	(14.3)
Christchurch	219	34	(15.5)	2	(0.9)	182	(83.1)	1	(0.5)

n, number of trips unless otherwise specified.

**Table 4:** Daily weather characteristics (n = 776)

	**North Shore City**		**Waitakere City**		**Wellington City**		**Christchurch City**		**All cities combined**	
	**Mean**	**(SD)**	**Mean**	**(SD)**	**Mean**	**(SD)**	**Mean**	**(SD)**	**Mean**	**(SD)**
Total hours sunlight	5.3	(3.5)	5.7	(3.0)	6.7	(4.5)	5.2	(4.4)	5.7	(4.0)
Average temperature	13.8	(2.4)	12.7	(3.5)	13.9	(3.7)	12.4	(4.3)	13.2	(3.6)
Total rainfall (mm)	3.4	(7.1)	2.9	(6.2)	1.3	(2.8)	1.4	(3.8)	2.2	(5.3)
Total rainfall, *n (%) days*										
None	84	(42.4)	74	(43.5)	121	(64.0)	145	(66.2)	424	(54.6)
Some	114	(57.6)	96	(56.5)	68	(36.0)	74	(33.8)	352	(45.4)

n, number of days; SD, standard deviation.

Values are Mean (SD) unless noted otherwise.

Child participant characteristics are outlined in Table 1. Children were aged between 6.5 and 15.0 (mean 11.6, SD 2.1) years. A majority of participants were classified as being of Māori, Asian, or New Zealand European ethnicity; the small number of participants who reported otherwise were grouped into a New Zealand European/other category.

Female sex (p = 0.10), child age (p = 018), city (p = 0.07), ethnicity (p = 0.12), living in a household with a higher household income (p = 0.02), residing within zone of school attended (p = 0.09), shorter distance to school (p < 0.001), NHSS^OTH^ (p = 0.08), city (p = 0.07), and sunlight hours (p = 0.16) all had p-values of < 0.20 in the bivariate analyses and so were simultaneously considered in a multivariate model (Table 5). Following backwards elimination of non-significant factors (p > 0.05) in the multivariate model, shorter distance to school (p < 0.001), child age (p = 0.005), city (p = 0.03), and NHSS^OTH^ (p = 0.04) remained significantly associated with likelihood of undertaking ATS. Accounting for age, city, and NHSS status, those living further than 2 km from school were significantly less likely to undertake ATS than those residing 700 m or less from school (OR 0.02, 95% CI 0.003, 0.10). Accounting for distance to school, city, and NHSS status, children of intermediate and secondary school age were significantly more likely to undertake ATS than their younger counterparts (OR 3.44, 95% CI 1.31, 9.01 and OR 2.88, 95% CI 1.15, 7.22, respectively). Taking distance to school, child age, and city into account, those children residing in a low walkable area and whose parents preferred a high walkable neighbourhood were 3.02 times less likely to use ATS than their counterparts (95% CI 1.07, 8.51). Finally, taking distance to school, child age, and NHSS status into account, significant differences were observed in ATS prevalence between cities whereby children residing in North Shore City were approximately twice as likely to use ATS than children residing in other cities. Compared with children living in North Shore City, children residing in Christchurch had the lowest odds of undertaking ATS (OR 0.23, 95% CI 0.08, 0.72).

**Table 5:** Results from bivariate and multivariate generalised estimation equation regressions of children’s active transport to school against potential predictor variables

	**Child active transport to school**					
	**Bivariate analyses**			**Final multivariate model**^**†**^		
	**OR**	**(95% CI)**	**p-value**	**OR**	**(95% CI)**	**p-value**
Age (years)			0.18*			0.005
5-10	1.00	Reference		1.00	Reference	
11-12	1.72	(0.72, 4.12)		3.44	(1.31, 9.01)	
13-14	1.59	(0.70, 3.59)		2.88	(1.15, 7.22)	
Sex			0.10*			
Male	1.00	Reference				
Female	1.79	(0.89, 3.60)				
Ethnicity			0.12*			
Māori	1.00	Reference				
Asian	3.05	(0.91, 10.23)				
New Zealand European/other	1.55	(0.59, 4.06)				
Average annual household income (NZD)			0.02*			
Lower (0-$80,000)	1.00	Reference				
Higher ($80,001+)	2.39	(1.13, 5.05)				
Car access			0.23			
Unlimited/frequent	1.00	Reference				
Limited/none	2.04	(0.64, 6.50)				
School zoning			0.09*			
Residing in school zone	1.00	Reference				
Residing outside school zone	0.32	(0.09, 1.20)				
Distance to school			<0.001*			<0.001
0-700 m	1.00	Reference		1.00	Reference	
701-1000 m	0.20	(0.07, 0.52)		0.17	(0.06, 0.48)	
1001-2000 m	0.38	(0.15, 0.94)		0.34	(0.13, 0.92)	
>2000 m	0.02	(0.004, 0.14)		0.02	(0.003, 0.10)	
Neighbourhood self selection			0.08*			0.04
Prefer high walkable, live low walkable	1.00	Reference		1.00	Reference	
Other	2.21	(0.91, 5.35)		3.02	(1.07, 8.51)	
City^#^			0.07*			0.03
North Shore	1.00	Reference		1.00	Reference	
Waitakere	0.81	(0.33, 2.02)		0.51	(0.18, 1.42)	
Wellington	0.40	(0.15, 1.06)		0.47	(0.13, 1.64)	
Christchurch	0.33	(0.13, 0.85)		0.23	(0.08, 0.72)	
Rainfall			0.21			
None	1.00	Reference				
Some	1.13	(0.93, 1.36)				
Sun hours	1.02	(0.99, 1.04)	0.16*			
Average temperature	1.01	(0.97, 1.04)	0.72			

*Wald’s p < 0.20 and entered into multivariate model.

^#^Fixed effect in all bivariate and multivariate models.

^†^p-value for final multivariate model <0.0001.

Notes: Total number of participants = 156, total number of observations = 748, with the exception of missing participant data for sex (n = 1), body mass index (n = 1), and school zoning (n = 18).

CI, confidence interval; m, metres; NZD, New Zealand Dollars; OR, odds ratio.

## Discussion

The aim of this study was to examine factors associated with ATS. including, for the first time, NHSS status. This was also the first study to consider ATS and weather patterns as daily repeated measures, improving sensitivity and modelling robustness. Results showed a significant association between NHSS status and ATS, whereby children who lived in a low-walkable neighbourhood, but whose parents preferred a highly walkable neighbourhood (NHSS^PHLL^) were three times less likely to use ATS than their counterparts (NHSS^OTH^). In other words, children residing in a highly walkable neighbourhood (irrespective of parental neighbourhood preference) or those residing in a low-walkable neighbourhood whose parents preferred a low-walkable neighbourhood, were significantly more likely to use ATS. The latter may be indicative of an issue of socio-economic status, whereby a “match” in neighbourhood walkability and preference was indicative of a family’s ability to afford to live in a neighbourhood of their choosing. The former supports adult research that shows the positive influence of neighbourhood walkability on active transport behaviours. NHSS has explained approximately 42% of differences in latent modelling of adult vehicle miles travelled between similar households living in urban/more walkable versus rural/less walkable neighbourhoods [57]. Likewise, preferring and residing in a more walkable neighbourhood was associated with active transport in a large sample of New Zealand adults [49]. No other comparable examinations for children exist.

As observed in previous research [58], no relationship was found between daily weather patterns and ATS. The dichotomisation of rainfall as none versus some may have hindered our ability to detect any association between substantial rainfall and ATS. Due to the nature of the rainfall data however, this approach was necessary to ensure that modelling remained robust. Moreover, previous research has shown that even when comparing days with no versus some rain, significant differences in physical activity levels can be found in children [37,38]. ATS may be less amenable to temporal factors such as weather and determined predominantly by pre-existing built environment and social variables such as time and convenience [24]. While improving on earlier research that has considered seasonality or weekly weather patterns in relation to activity, the use of a daily measure of weather data may still have been insufficiently sensitive to identify relationships between ATS and weather patterns. Moreover, weather data were captured from one primary weather station for each respective city. As such, differences existed in distance to these weather stations across and within cities. Therefore, it is possible that differential weather patterns were observed for neighbourhoods and individuals within each city and so the association between weather factors and ATS may have been diluted accordingly. Future research should consider better spatio-temporal matching of weather exposures for individuals. For example, this might involve extracting weather data for periods of the day where ATS might be expected to occur, and undertaking measures of weather at finer spatial resolutions (e.g., at the school or neighbourhood, rather than city, level). It is possible, however, that decision making regarding travel mode is not limited to exact temporal or spatial exposure. For example, predicted weather patterns or heavy rainfall in the early morning may influence travel plans for later periods of the day, irrespective of actual weather at the time of the journey.

Significant differences were observed in ATS between cities, with children residing in North Shore City approximately twice as likely to use ATS than their counterparts living in other cities. These findings conflict with national prevalence data for New Zealand that suggests a greater proportion of trips are made by walking or cycling in Christchurch and Wellington Cities than in Auckland City (4%, 3%, and 2% of kilometres travelled per person (children and adults), per year, respectively) [11]. Reasons for this finding are unclear; it is possible that local initiatives such as the Travelwise school travel plan programme, initiated in North Shore City prior to data collection for the current study, may have influenced children’s travel behaviours in this region [59]. It is also possible that variables such as home ownership and length of residency may reflect a greater ability to ‘self select’ a neighbourhood, and that these variables differed between cities, however we were unable to assess these differences in the current investigation.

Household income was not significantly related to ATS after accounting for other factors in the multivariate modelling. Earlier New Zealand research has shown that children residing in high deprivation areas are more likely to use ATS than those living in the least deprived areas [60]. However, internationally, research investigating associations between socio-economic status and ATS has been equivocal, with positive, negative, and insignificant relationships found [26,61]. Similarly, after accounting for other significant factors from the bivariate analyses, we found no relationship between car access (or lack thereof) and ATS in the current study. Nearly all (90%) respondents had frequent or unlimited car access, thus homogeneity in this factor may have hindered our ability to detect a relationship with ATS [62].

In keeping with previous research, our findings showed increasing distance to school was significantly related to a reduced likelihood of ATS [30,63]. A substantial drop in prevalence of ATS was seen even for those children who lived further than 700 m from school. It is worth noting that almost all of the ATS observed in the current study was via walking. A study with parents of Belgian youth aged 11–12 years suggested that criterion distances of 1.5 km and 3.0 km are optimal for encouraging ATS via walking and cycling respectively [28], however whether these findings hold true for other populations remains to be determined. School catchment zones vary widely in New Zealand (up to 90 km using a Euclidean diameter). US data suggest that only 20% of children live within 1.6 km (1 mile) from school [32]. Even so, for children who do live within this distance, surveys have shown that a high proportion of children do not actively commute to school [32,64]. A number of Australian studies exemplify the discrepancies between residing close enough to school for children to use active transport despite little uptake of active travel modes. Parents of children aged 5–6 and 10–12 years identified a walking distance of 800 m in one direction as being appropriate for their children, roughly equivalent to a 15-minute walk [65]. Despite this observation, a later study of 4–13 year old children found that while over half of the participants lived within a 15 minute walk to school, parents still reported their child’s school was too far away to reach by walking [66]. Yet another study showed that of children living within 400 metres of school, 21% were still driven by car, even though trip durations by car or walking were strikingly similar (mean duration of 8 and 7 minutes, respectively) [67].

Cumulatively, these results suggest that localised schools nested within communities may facilitate increased uptake of ATS. Irrespective of actual school zoning, recent trends in school siting and consequent effects on upsizing have created a significant barrier to children actively travelling to and from school. For example, student numbers in the US have grown, yet the number of small local schools has dropped and there has been a consequent increase in ‘supersized’ schools that service a wider geographic spread [68]. When considering school siting, it may also be important to take into account other factors such traffic volume, which may mediate or moderate the positive effect of street connectivity on ATS [23].

Age was significantly related to ATS, whereby children aged 11–14 years (reflecting intermediate and secondary school ages) were approximately three times more likely to use ATS than their younger counterparts (aged 5–10 years). Although contradictory findings have been reported regarding school travel and age [30], our findings are in keeping with those from the national New Zealand Travel Survey, which show a greater prevalence of walking and cycling for transport in youth aged 13–17 years (31%), compared with children aged 5–12 years (29%) [69]. All other factors being equal, an increase in ATS with age/school level is unsurprising, and may be indicative of increasing parental licence, whereby older children have more freedom to travel independent of adult supervision [70].

Aside from NHSS, parental factors such as safety concerns (e.g., about crime, traffic, sidewalks and cycle lanes/bikeways), supports for ATS, and factors influencing these were not examined. As such, we cannot determine the relative contribution that distance to school has above and beyond these parental factors, which may also be independently associated with ATS, or moderate or mediate the relationships found [22,30,33]. We also focused on the trip to school only, a pragmatic choice based on the expectation of less trip-chaining on the trip to school [24], as recently evidenced in a study of independent mobility in New Zealand children [71].

## Conclusion

We present the first examination of the relationship between NHSS status and weather patterns with children’s ATS, using daily ATS behaviours and weather as repeated measures. Our findings support previous research that distance to school is the prevailing environmental factor associated with children’s ATS. This study, in conjunction with previous research, suggests that school siting is likely an important associate of ATS in children. Localised interventions that support ATS in primary school-aged children in particular may also be worthwhile in the New Zealand context. Current urban developments will have long-lasting effects on active transportation uptake and associated outcomes; this research contributes to the evidence base for environmental planning and intervention development for improving ATS uptake in children.

## Competing interests

The authors declare that they have no competing interests.

## Authors’ contributions

MO drafted the manuscript and conducted the data analyses. HB, KW, and RK participated in the design of the URBAN study and provided input specifically for the current investigation regarding neighbourhood self-selection, social and physical environmental factors, and children’s school travel behaviours, respectively. SM prepared and analysed all geographic information systems data. AE provided input and advice regarding children’s travel behaviours. EH contributed to the draft manuscript. LM and HB managed the URBAN database; LM conducted data cleaning, reduction, and preparation for the current investigation. PS provided biostatistical support and assistance with data analyses. All authors contributed to the draft manuscript, and read and approved the final manuscript.
